# Nutritional Status and Chemotherapy Completion in Resectable Pancreatic Cancer: A Narrative Review

**DOI:** 10.3390/curroncol32090519

**Published:** 2025-09-17

**Authors:** Naotake Funamizu, Mio Uraoka, Chihiro Ito, Miku Iwata, Akimasa Sakamoto, Yoshiaki Kamei, Yuzo Umeda

**Affiliations:** Department of Hepatobiliary Pancreatic and Breast Surgery, Graduate School of Medicine, Ehime University, Shitsukawa 454, Toon-City 791-0295, Ehime Prefecture, Japan; uraoka.mio.lr@ehime-u.ac.jp (M.U.); chippy.ito@gmail.com (C.I.); miku.nkgw@gmail.com (M.I.); sakamoto.akimasa.kw@ehime-u.ac.jp (A.S.); kamei.yoshiaki.mz@ehime-u.ac.jp (Y.K.); umeda.yuzo.oe@ehime-u.ac.jp (Y.U.)

**Keywords:** pancreatic cancer, perioperative chemotherapy, nutritional status, chemotherapy completion, dysbiosis

## Abstract

Pancreatic cancer (PC) remains associated with poor survival despite curative-intent resection. Many patients fail to complete the planned course of adjuvant chemotherapy (AC), most often due to early postoperative nutritional decline. We conducted a narrative review (PubMed, EMBASE, Scopus; Jan 2010–Dec 2024; last update 15 February 2025) focusing on two endpoints: (1) nutritional predictors of completing ≥80% of planned AC cycles and (2) optimal timing of perioperative nutritional interventions. Preoperative GNRI and PNI, early postoperative albumin and CAR, and sarcopenia are robust predictors of AC adherence and survival. Nutritional decline is frequently accelerated by systemic inflammation and pancreatic exocrine insufficiency. Observational data indicate that interventions initiated within 2–4 weeks after surgery—before starting AC—improve chemotherapy completion rates, especially when guided by dynamic monitoring of nutritional and inflammatory markers. Neoadjuvant chemotherapy (NAC) offers a potential window for metabolic optimization, but validated nutrition-based predictors of NAC adherence remain limited. Readily measurable nutritional indices such as PNI, GNRI, and CAR should be integrated into early perioperative assessments to identify high-risk patients. Timely, phase-specific interventions, including enzyme replacement and anti-inflammatory nutritional strategies, can enhance AC adherence and improve prognoses. These findings support the transition from a supportive to a proactive, personalized nutritional oncology model in resectable PC.

## Simple Summary

Pancreatic cancer (PC) is highly lethal, and many patients do not complete postoperative adjuvant chemotherapy (AC). Emerging evidence shows that simple nutritional indices—the prognostic nutritional index (PNI), the geriatric nutritional risk index (GNRI), and the C-reactive protein/albumin ratio (CAR)—predict AC completion and survival across commonly used regimens (modified FOLFIRINOX, gemcitabine–capecitabine, and regionally adopted S-1). In PC, early postoperative declines in nutritional status, driven by systemic inflammation, sarcopenia, and pancreatic exocrine insufficiency, often interrupt treatment. This review focuses on two clinical questions in PC: which nutritional markers predict completing ≥80% of planned AC cycles and when to start nutritional intervention. We highlight recent data showing that interventions within 2–4 weeks after surgery, guided by nutritional and inflammatory markers, may improve chemotherapy adherence and outcomes. Embedding these assessments into standard perioperative care could move nutrition from a supportive role to a proactive, central component of personalized oncology.

## 1. Introduction

In this review, we define “malnutrition” according to the Global Leadership Initiative on Malnutrition (GLIM) criteria (phenotypic and etiologic components) and “cachexia” as a multifactorial syndrome characterized by progressive skeletal muscle loss—unresponsive to conventional nutrition—and systemic inflammation (e.g., >5% weight loss over 6 months or body mass index (BMI) < 20 kg/m^2^ with weight loss > 2%, plus elevated inflammatory markers) [1,2,3]. Pancreatic cancer (PC) remains one of the most lethal malignancies, with an estimated 66,440 new cases and 51,750 deaths projected in the United States for 2024 [4]. Global data show similarly poor outcomes, with 5-year survival rates generally below 12% in Western countries and only modestly higher in regions where adjuvant chemotherapy (AC) including modified FOLFIRINOX (folinic acid [leucovorin], 5-fluorouracil, irinotecan, and oxaliplatin), gemcitabine–capecitabine, and S-1, is widely adopted. Nearly 70% of patients are already malnourished at diagnosis, driven by tumor-induced inflammation, pancreatic exocrine insufficiency, and treatment-related gastrointestinal toxicity [5,6,7]. These nutritional deficits impair chemotherapy tolerance, compromise immune competence, and accelerate functional decline. Consequently, malnutrition has been consistently linked to postoperative complications (POCs), early discontinuation of adjuvant chemotherapy (AC), and poor prognosis [8,9,10,11].

Recognizing this, the National Comprehensive Cancer Network (NCCN) Clinical Practice Guidelines in Oncology: Pancreatic Adenocarcinoma (Version 2.2025) recommend routine perioperative nutritional screening using validated assessment tools, alongside institution-wide implementation of tailored interventions such as high-protein diets, pancreatic enzyme replacement therapy (PERT), and structured physical activity programs [12]. While the guidelines do not specify indices, tools such as the Geriatric Nutritional Risk Index (GNRI) and Prognostic Nutritional Index (PNI) are widely used in clinical research to stratify nutritional risk in pancreatic cancer patients. Similarly, the 2023 European Society for Clinical Nutrition and Metabolism (ESPEN) consensus statement emphasizes that nutritional care should start at diagnosis, be maintained throughout the perioperative course, and be adapted according to ongoing assessments of nutritional risk and functional status [13].

The survival benefits of adjuvant therapy are well established internationally. Randomized trials, including CONKO-001 [14], PRODIGE 24/CCTG PA.6 and ESPAC-4, have established modified FOLFIRINOX and gemcitabine–capecitabine as widely used standards [15,16], while S-1 is adopted in certain East Asian settings based on JASPAC-01 [17]. Our focus in this review is not to compare regimens but to synthesize how nutritional status influences the completion of adjuvant chemotherapy across regimens. In the Prep-02/JSAP-05 trial, neoadjuvant chemotherapy (NAC) with gemcitabine plus S-1 before surgery yielded a 28% reduction in the risk of death and superior 2-year survival compared with upfront surgery [18]. Nevertheless, postoperative nutritional decline—manifested by hypoalbuminemia, elevated C-reactive protein-to-albumin ratio (CAR), and marked weight loss—remains a major barrier to AC completion [19]. Accordingly, AC should be initiated as early as clinically feasible once patients are medically fit; exact timing varies across guidelines and regimens. We therefore frame the “pre-AC nutritional optimization” window pragmatically rather than anchoring it to a single trial. This timeframe serves as the target for pre-AC nutritional optimization discussed in this review [17]. Large-scale observational cohorts and meta-analyses confirm that low GNRI or PNI and high CAR predict chemotherapy non-adherence and inferior survival [20,21,22,23,24]. Despite this, nearly half of malnourished patients receive no structured nutritional support [25], often due to limited oncologic awareness, insufficient dietetic staffing, and lack of multidisciplinary coordination. A range of interventions has demonstrated potential benefit. PERT mitigates malabsorption and weight loss [26], and has been associated with improved overall survival in observational cohorts [27,28] in patients with pancreatic exocrine insufficiency. Prehabilitation and rehabilitation programs that combine individualized nutrition counseling, resistance exercise, and anti-inflammatory dietary components (such as ω-3 fatty acids) can improve inflammatory–nutritional markers and functional capacity [29,30,31]. Expert panels, including the International Study Group on Pancreatic Surgery, emphasize the importance of longitudinal monitoring of weight, skeletal muscle mass, and overall nutritional status throughout the perioperative period [32].

**Figure 1:**
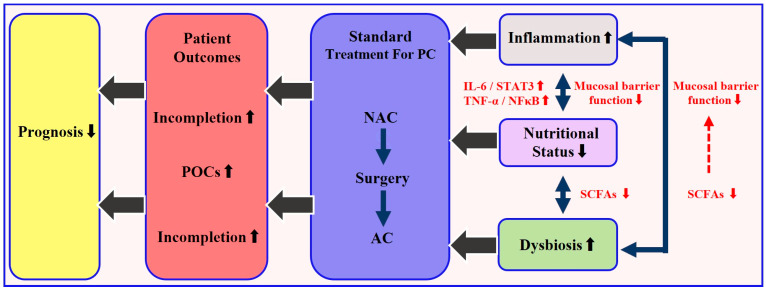
Schematic of the microbiota–nutrition–inflammation axis in resectable pancreatic cancer and its effect on the treatment sequence (NAC → surgery → AC). Dysbiosis reduces short-chain fatty acid (SCFA) production and impairs the barrier function, leading to malabsorption and malnutrition. This triggers excess IL-6 and TNF-α, activating JAK/STAT3 and NF-κB pathways, which worsen systemic inflammation and muscle loss. The cycle increases postoperative complications and lowers chemotherapy completion rates, leading to poorer prognosis. Note: For resectable PDAC, neoadjuvant chemotherapy (NAC) is not universally standard and remains under investigation; the sequence shown is a schematic for discussing nutrition and does not imply guideline uniformity.

Mechanistic insights provide further rationale for targeted nutritional strategies. Pro-inflammatory cytokines, particularly IL-6 and TNF-α, mediate muscle proteolysis and adipose tissue browning via the JAK/STAT3 and NF-κB pathways, thereby exacerbating cachexia and reducing chemotherapy tolerance [33,34]. In parallel, gut microbiota dysbiosis diminishes production of beneficial short-chain fatty acids (SCFAs), compromising intestinal barrier function and amplifying systemic inflammation. Butyrate, a key SCFA, has been shown to inhibit histone deacetylase 8 (HDAC8), suppress NF-κB–driven pro-inflammatory genes, and up-regulate tight junction proteins, leading to improved nutrient absorption and reduced chemotherapy-induced mucositis [35]. These microbiota–nutrition–inflammation interactions, illustrated in Figure 1, underscore the potential of integrating biomarker-guided nutritional support into standard perioperative care (Figure 1).

Collectively, these data highlight a dual challenge: identifying patients at highest nutritional risk before and after surgery, and delivering timely, evidence-based interventions to maintain or restore their capacity to complete planned chemotherapy. This review focuses on two clinically actionable endpoints: (1) nutritional determinants of completing ≥80% of planned AC cycles, and (2) optimal timing of nutritional intervention. In doing so, we aim to provide a practical framework that synthesizes current evidence, acknowledges existing limitations, and outlines priorities for future clinical trials in personalized nutritional oncology. Accordingly, when regimen-specific studies are cited—many of which come from East Asia using S-1—the biological rationale and observed associations (e.g., with GNRI, PNI, CAR, sarcopenia) are interpreted as regimen-agnostic unless otherwise specified.

## 2. Materials and Methods

We performed a comprehensive literature search in PubMed, EMBASE, Scopus, and the Cochrane Library (January 2010–December 2024; last update 15 February 2025) using both controlled vocabulary (e.g., MeSH) and free-text terms for “pancreatic adenocarcinoma” or “resectable pancreatic cancer” and nutritional parameters (GNRI, PNI, CAR, sarcopenia, serum albumin). Boolean operators and filters for English language and human studies were applied. The PubMed search string was: (“pancreatic adenocarcinoma” [MeSH] OR “resectable pancreatic cancer”) AND (“Geriatric Nutritional Risk Index” OR “GNRI” OR “Prognostic Nutritional Index” OR “PNI” OR “C-reactive protein-to-albumin ratio” OR “CAR” OR “sarcopenia” OR “serum albumin”). Database-specific adaptations were used for EMBASE, Scopus, and Cochrane Library.

We included adult patients (≥18 years) with resectable PC receiving NAC and/or AC and reporting at least one nutritional index or intervention, with outcomes related to chemotherapy completion, tolerance, or survival. We a priori defined “chemotherapy completion” as maintaining a relative dose intensity (RDI) ≥ 80% over the planned 6-month AC course; when only cycle counts were reported, completion of ≥80% of planned cycles was considered an approximate equivalent endpoint. Exclusion criteria comprised case reports, editorials, letters, conference abstracts, non-original works, non-English publications, and studies without full text.

**Figure 2:**
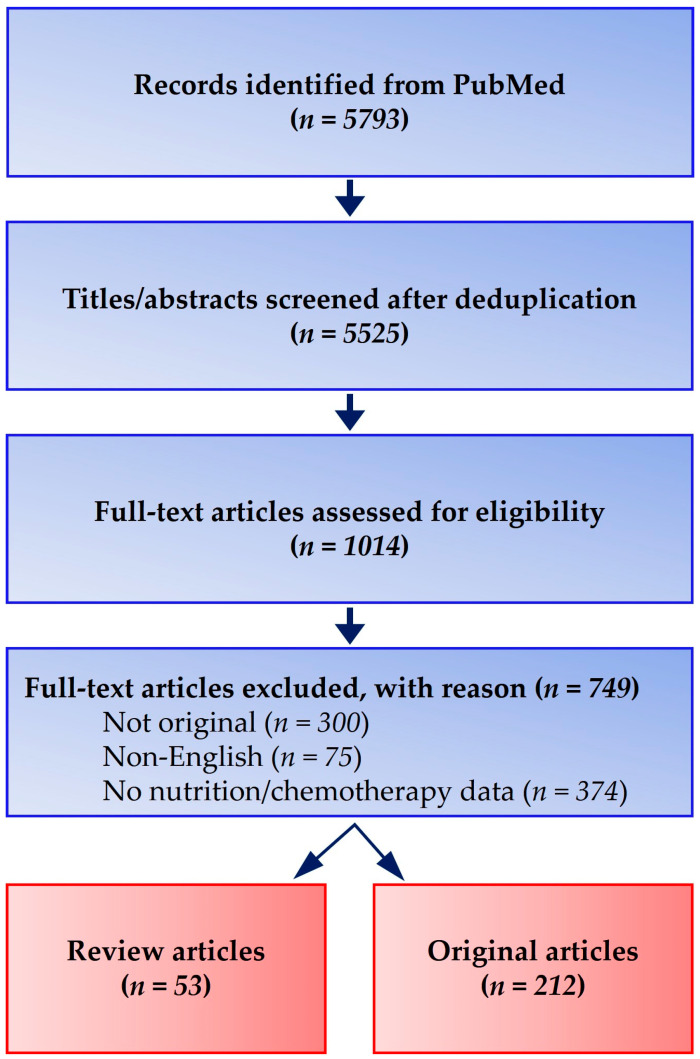
PRISMA-style flow diagram of literature identification and selection. A comprehensive search (January 2010–December 2024; final update 15 February 2025) in PubMed, EMBASE, Scopus, and the Cochrane Library retrieved 5793 records. After deduplication (*n* = 5525) and screening, 1014 full-text articles were assessed, with 212 original studies and 53 review articles meeting inclusion criteria.

Screening followed a Preferred Reporting Items for Systematic Reviews and Meta-Analyses (PRISMA)-style flow: of 5793 records retrieved, 5525 remained after deduplication; 1014 full texts were reviewed, and 212 original studies plus 53 review articles were included. Discrepancies between two independent reviewers were resolved by consensus (Figure 2).

Limitations include the narrative nature of this review, potential selection and publication bias, and reliance primarily on PubMed, although supplemental searches in other databases were conducted to minimize omissions. Greater weight was given to prospective, multicenter studies with external validation.

**Evidence appraisal.** We assigned study-level designs to the Oxford Centre for Evidence-Based Medicine (OCEBM) 2011 Levels of Evidence according to the clinical question (therapy vs. prognosis) [36]. We then rated outcome-level certainty using GRADE (high, moderate, low, very low), considering risk of bias, inconsistency, indirectness, imprecision, and publication bias; upgrading criteria were applied when appropriate (large effect, dose–response, residual confounding) [37,38]. Two reviewers assessed each item independently; discrepancies were resolved by consensus.

## 3. Results

### 3.1. Nutritional Predictors of AC Completion

Although several cohorts evaluated S-1 in East Asia, these indices have also been associated with adherence and outcomes in studies of modified FOLFIRINOX and gemcitabine-based AC; thus we consider them broadly applicable across regimens. Multiple clinical parameters reflecting nutritional and inflammatory status have been evaluated as predictors of AC completion in resectable PC. The most consistently validated are the GNRI, PNI, CAR, and sarcopenia.

- GNRI: Low preoperative GNRI is associated with early discontinuation of S-1 AC and reduced survival in multiple retrospective and prospective cohorts, including studies with external validation [20,21,39,40].
- PNI: Lower PNI values—calculated from serum albumin and lymphocyte count—predict incomplete AC and poorer survival [41,42,43,44].
- CAR: Elevated CAR, reflecting combined systemic inflammation and malnutrition, independently forecasts poor S-1 tolerability and inferior recurrence-free and overall survival [22,23,45,46].
- Sarcopenia: Skeletal muscle depletion predicts reduced chemotherapy tolerance and completion, as well as worse overall survival [47,48,49,50,51,52,53].

These indices outperform single variables such as BMI or albumin alone, as they integrate both nutritional and inflammatory dimensions. Table 1 summarizes the main indices and their clinical characteristics.

### 3.2. Neoadjuvant Chemotherapy (NAC) vs. AC: Nutritional Vulnerability

In contrast to AC, NAC is administered before postoperative nutritional decline and therefore generally achieves higher treatment completion rates. The Japanese randomized Prep-02/JSAP-05 trial, along with complementary cohort studies, demonstrated that gemcitabine plus S-1 NAC preserved nutritional status and improved resection outcomes [54,55]. While multi-agent regimens such as FOLFIRINOX are widely adopted in international practice, our nutritional framework is intentionally regimen-agnostic. By contrast, AC has demonstrated unequivocal survival benefits in landmark randomized controlled trials, including CONKO-001, ESPAC-4, and PRODIGE 24 [14,15,16,17]. However, AC delivery is particularly vulnerable to postoperative weight loss, sarcopenia, and hypoalbuminemia, which have been consistently associated with premature discontinuation of therapy [10,11]. These observations underscore the central role of nutritional status as a determinant of successful chemotherapy completion.

### 3.3. Timing of Nutritional Intervention

Evidence, though mostly observational, suggests that early postoperative nutritional intervention (within 2–4 weeks after surgery) improves AC completion and functional recovery compared with interventions starting at AC initiation. AC is ideally initiated once the patient is medically fit—commonly within ~12 weeks after surgery, per prevailing guidance—though trial-specific windows vary (e.g., ≤10 weeks in JASPAC-01 [17]). Therefore, we consider the “pre-AC nutritional optimization” period pragmatically, without anchoring it to any single study.

- PERT: Timely initiation after pancreaticoduodenectomy prevents malabsorption, limits weight loss, and has been associated with improved survival [26,27,28].
- High-protein, high-calorie diets and immunonutrition: Use of omega-3 fatty acids and other immunonutrition components during the early postoperative period may help preserve lean mass and reduce inflammation [56,57,58,59,60,61].
- Prehabilitation programs: During NAC, combined nutritional counseling and exercise interventions have demonstrated improvements in PNI and inflammatory ratios before surgery [31,62,63].

**Figure 3:**
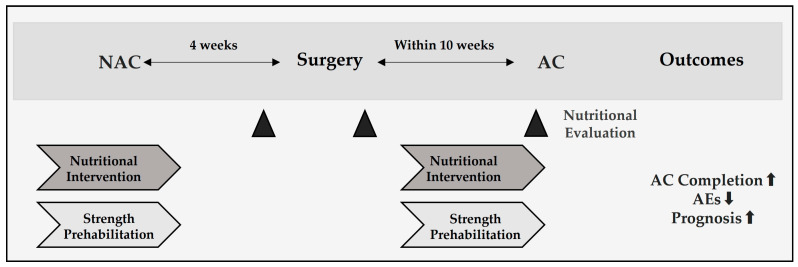
Perioperative timeline and recommended windows for nutritional evaluation and intervention in resectable pancreatic cancer. Neoadjuvant chemotherapy (NAC) is followed by surgery at approximately 4 weeks, and adjuvant chemotherapy (AC) should begin early after surgery once patients are clinically ready, in accordance with prevailing guidelines and institutional practice. Nutrition checkpoints at the end of NAC, postoperative day 7–14, and pre-AC assess GNRI/PNI, albumin/CAR, and sarcopenia to prompt a dietitian-led high-protein diet with or without oral nutritional supplements (ONS), pancreatic enzyme replacement (PERT), and resistance training—aiming to maintain AC relative dose intensity (RDI) at ≥80% for 6 months.

Table 2 presents interventions stratified by treatment phase (NAC, early postoperative, AC). Figure 3 outlines the potential impact of intervention timing across the perioperative course.

### 3.4. Limitations of Current Evidence

Most studies are retrospective single-center analyses; randomized controlled trials specifically designed to test nutritional optimization for AC completion in PC are lacking. Predictors of NAC adherence are less well-validated than those for AC. Multicenter prospective trials are needed to confirm optimal intervention timing and content.

Interventions include nutritional counseling, pancreatic enzyme replacement therapy (PERT; for pancreatic exocrine insufficiency [PEI]), immunonutrition, exercise therapy, and emerging options such as vitamin D analogs. Their timing and impact vary depending on the treatment context—neoadjuvant, postoperative, or adjuvant; where pancreatic-specific trials are limited, evidence from gastrointestinal (GI) cancers is cited.

## 4. Discussion

### 4.1. Clinical Evidence Linking Nutritional Status and Chemotherapy Outcomes with Nutritional Indices and Sarcopenia

Historically, composite nutritional–inflammatory indices, such as GNRI, CAR, and PNI, together with sarcopenia, were primarily validated as predictors of postoperative outcomes and survival after pancreatic resection [65,66], with additional risk conveyed by the combined effect of frailty and sarcopenia [67,68]. More recently, multi-institutional cohorts with external validation have demonstrated that these indices also predict adherence to AC and early discontinuation, refining pre-AC risk appraisal alongside sarcopenia [20,22,67]. Taken together, because these indices capture inflammation-nutrition biology rather than drug-specific effects, their predictive relevance extends to commonly used international adjuvant regimens (e.g., modified FOLFIRINOX, gemcitabine–capecitabine)

#### 4.1.1. GNRI (Geriatric Nutritional Risk Index)

A lower preoperative GNRI predicts POCs after resection and early discontinuation of S-1 AC after curative surgery in a multi-institutional study with external validation, supporting GNRI as a practical pre-AC triage tool [19,20,69].

#### 4.1.2. CAR (C-Reactive Protein-to-Albumin Ratio)

A higher preoperative CAR is associated with POCs after resection, and poor tolerability and early cessation of S-1 AC after curative resection, shown in a development cohort and confirmed by external validation [21,22,70].

#### 4.1.3. PNI (Prognostic Nutritional Index)

In patients who received NAC followed by curative surgery, PNI < 45 independently predicted failure to continue S-1 AC, and meta-analytic data link low preoperative PNI with worse post-resection survival—supporting its use alongside GNRI/CAR for pre-AC risk appraisal [8,42,43].

#### 4.1.4. Sarcopenia

Sarcopenia. CT-defined sarcopenia (low skeletal muscle index at L3) has been associated with treatment modifications and failure to complete S-1 AC in PC cohorts; moreover, in patients receiving adjuvant modified FOLFIRINOX or gemcitabine, baseline sarcopenia was linked to markedly shorter overall survival irrespective of regimen, underscoring its prognostic and tolerance implications during AC [71]. Sarcopenic obesity and skeletal muscle depletion strongly predict outcomes independent of BMI, aligning with our emphasis on body-composition–aware indices [72,73,74].

### 4.2. Nutritional Interventions and Their Protective Effects

Targeted nutritional interventions have the potential to modify treatment trajectories in resectable PC. In clinical settings, early, structured nutritional support during chemotherapy has been associated with better treatment adherence and survival in PC cohorts [75]. Clinically, individualized nutritional counseling and oral nutritional supplements (ONS) have been associated with improved treatment tolerance, quality of life, and AC completion [76,77]. Perioperative or chemotherapy-concurrent omega-3–enriched nutrition has shown reductions in CRP and signals for improved tolerance in clinical studies of GI/pancreatic cancer, including a randomized phase II trial in advanced PC (gemcitabine ± EPA); however, effects on lean mass, survival, and AC completion remain heterogeneous across trials [57,59,60,61].

Despite these data, malnutrition and cachexia are often underdiagnosed, and nutritional therapy remains underutilized in oncology practice. Early and sustained implementation of evidence-based nutritional support—tailored to individual inflammatory and metabolic profiles—should be viewed as a proactive, modifiable component of perioperative care. In metastatic PDAC, early nutritional support has been associated with improved treatment tolerance and patient-centered outcomes, although prospective evidence remains limited [25,78].

### 4.3. Mechanistic Insights: Inflammation, Muscle Proteolysis, and Immune Modulation

Preclinical studies have elucidated the molecular basis by which nutritional decline compromises treatment tolerance. Interleukin-6 (IL-6) family cytokines activate the JAK/STAT3 pathway via the IL-6Rα/gp130 receptor complex, thereby inducing acute-phase protein synthesis, skeletal muscle proteolysis, and browning of adipose tissue—core phenotypic features of cancer cachexia [33]. These processes lead to muscle weakness and diminished physiological reserve, ultimately reducing tolerance to cytotoxic therapies. In parallel, tumor necrosis factor-α (TNF-α) engages TNFR1/2 to activate NF-κB signaling, which upregulates muscle-specific E3 ubiquitin ligases such as MuRF1 and Atrogin-1, accelerating protein degradation through the ubiquitin–proteasome system [79]. These inflammatory cascades also contribute to immunosuppression within the tumor microenvironment, further attenuating therapeutic efficacy [80]. Consistently, PC harbors a tumor microbiome that shapes antitumor immunity and disease trajectory, with specific bacterial consortia associating with outcome and immune reprogramming in preclinical and translational studies [81,82], and intracellular, tumor–type–specific bacteria documented across malignancies [83].

From a translational perspective, mechanism-guided interventions that temper IL-6/JAK/STAT3 activity are being explored. Although direct evidence in pancreatic cancer is limited, observational data in other malignancies suggest that IL-6 receptor blockade with tocilizumab can reduce CRP, increase serum albumin, maintain body weight, improve symptoms, and prolong median overall survival in cachectic patients with advanced non-small-cell lung cancer [84], while tocilizumab plus corticosteroids yielded short-term benefits in cancer cachexia with systemic hyperinflammation [85]. Direct STAT3 inhibition (TTI-101) has also shown favorable safety and pharmacodynamic activity in a phase I study of advanced solid tumors [86]. These observations support a multimodal approach coupling anti-inflammatory pharmacotherapy with nutrition and exercise to bridge mechanistic insights and clinical outcomes.

### 4.4. Gaps Between Evidence and Clinical Implementation

International guidance already specifies what “good practice” should look like: the 2017 ESPEN expert recommendations and the 2021 ESPEN practical guideline call for routine screening with validated tools, early dietitian involvement, and protocolized nutrition pathways embedded across oncology services [13,87]. Despite this, malnutrition remains common and undertreated in real-world oncology: a multicentre audit of French comprehensive cancer centers documented high prevalence at presentation, while in pancreatic/periampullary cancer, early dietetic consultation correlated with improved survival [88,89]. These data frame the current gap between recommendations and practice in perioperative PC care. Despite strong evidence, the translation of nutritional screening and intervention into routine perioperative PC care remains inconsistent. International guidelines, including NCCN v2.2025, recommend early screening; indices such as GNRI and PNI are widely used in research and in this review [12], yet observational studies indicate that nearly half of malnourished patients receive no structured nutritional support [90]. Barriers include limited oncologic awareness, resource constraints, and the absence of standardized care pathways linking surgical, oncologic, and nutrition teams.

Pancreatic exocrine insufficiency (PEI) after pancreaticoduodenectomy further exacerbates postoperative malnutrition through fat malabsorption. Although PERT improves nutrient absorption, mitigates weight loss, and has been associated with survival benefits [27,28], it remains underprescribed in many centers. Recent evidence indicates that dose optimization of PERT is essential to mitigate muscle loss in advanced PC patients with PEI [91], and systematic reviews confirm the high prevalence of PEI after pancreatic resection, supporting the need for routine postoperative screening and treatment [92]. Bridging this evidence–practice gap requires institutional protocols that embed nutritional and enzymatic support into standard perioperative oncology workflows. Formalizing a ‘nutritional oncology board’ has been proposed to close implementation gaps between surgical, medical, and nutrition teams [93].

### 4.5. Cancer Cachexia as a Therapeutic Target

Cachexia in PC represents a systemic metabolic derangement driven by both tumor-derived and host inflammatory signals. Cytokines such as IL-6 and TGF-β1 activate muscle proteolysis and lipolysis, while modulating immune responses in ways that impair therapeutic efficacy [34,94]. Recent mechanistic work has shown that TGF-β1 induces a KLF10-dependent transcriptional program promoting muscle atrophy, and IL-6–mediated STAT3 activation further amplifies systemic inflammation [95,96].

In PC-specific models, tumor organoid–derived factors from cachectic patients have been shown to disrupt contractile smooth muscle cells, providing a mechanistic link between tumor-secreted mediators and gastrointestinal motility impairment [97]. Clinically, cachexia-associated inflammation, captured by indices such as the CAR and GNRI, predicts survival more accurately than conventional nutritional assessments in PC and other gastrointestinal tumors [98].

These mechanisms underscore why cachexia is not merely a symptom but a direct mediator of poor prognosis. Given its high prevalence and predictive value for AC intolerance, cachexia should be targeted as aggressively as other modifiable risk factors, combining anti-inflammatory nutritional strategies (e.g., omega-3 PUFAs, immunonutrition) with structured exercise and metabolic support. Narrative and mechanistic overviews emphasize cachexia as a systemic, treatable driver of poor outcomes, not merely a comorbidity [99,100,101].

### 4.6. Toward Personalized Nutritional Oncology

While indices such as the GNRI, PNI, CAR, and sarcopenia are valuable for risk stratification, they do not fully capture the multidimensional determinants of treatment response. Integrating molecular and microenvironmental factors—such as microRNA profiles, epithelial–mesenchymal transition (EMT) status, and stromal activation markers—could refine predictive accuracy. For example, microRNAs such as miR-301b and miR-200b have been implicated in gemcitabine resistance through EMT promotion and tumor microenvironment modulation [102,103,104,105,106]. Experimental evidence also shows that deregulation of microRNA networks following key tumor suppressor loss, such as Ink4a/Arf, can further drive oncogenic transformation and treatment resistance [107]. Nutritional factors, notably vitamin D signaling, can counteract these resistance mechanisms by reprogramming pancreatic stellate cells, normalizing stromal architecture, and modulating immunosuppressive niches [64]. Such interactions suggest that future predictive models should combine inflammatory–nutritional indices with molecular resistance markers to guide both systemic therapy and supportive care.

### 4.7. Future Research Directions

Host–microbiome–tumor interactions are increasingly recognized as modifiers of both efficacy and toxicity of systemic cancer therapies beyond immune checkpoint inhibitors [108,109,110]. Mechanistically, SCFAs—particularly butyrate—can influence epithelial barrier integrity and inflammatory signaling through HDAC/NF-κB axes [35], providing a biologic rationale for integrating microbial metabolites into perioperative supportive care for PC.

Emerging synthesis work specific to PC indicates that microbially derived metabolites may mitigate chemotherapy resistance by rewiring tumor–stromal crosstalk and inflammatory pathways [111]. Complementing this metabolite-centric view, intratumoral microbiota can directly alter drug pharmacology: Geller et al. demonstrated that tumor-resident bacteria possessing cytidine deaminase activity can inactivate gemcitabine, thereby diminishing cytotoxicity [112]. Together, these observations support a development strategy that (i) profiles gut and intratumoral microbiomes and metabolites before NAC/AC, (ii) pilots selective antimicrobial, pre/pro/synbiotic, or dietary interventions, and (iii) quantifies tumor microbial drug-modifying enzymes (e.g., cytidine deaminase) in resection specimens.

For fluoropyrimidines, the S-1 combination (tegafur/gimeracil/oteracil) controls systemic 5-FU exposure chiefly via gimeracil-mediated inhibition of host dihydropyrimidine dehydrogenase (DPD). The gut microbiome may modulate fluoropyrimidine pharmacodynamics and mucosal toxicity through pyrimidine metabolism, bile-acid/SCFA signaling, and inflammation [108]. These considerations motivate an integrative monitoring framework—host DPD activity × microbiome/metabolites × inflammatory–nutritional indices—to personalize S-1 dosing and supportive care. Practically, the early postoperative “window” (2–4 weeks after resection) is well suited to implement: (a) concurrent assessment of SCFAs, bile acids, CRP/CAR, and GNRI/PNI; (b) small, adaptive trials of targeted pre/pro/synbiotics [27] with metabolite-response readouts; and (c) longitudinal tracking after S-1 initiation to sustain RDI ≥ 80% across the planned 6-month course.

Key design elements for future perioperative trials include:

1. Stratification: Baseline GNRI/PNI/CAR combined with microbial diversity and metabolite panels (SCFAs, TMAO).
2. Endpoints: Primary—maintenance of RDI ≥ 80% (6 months); secondary—completion of ≥80% of planned cycles, CTCAE-graded toxicities, QoL.
3. Testable interventions: Optimization of PERT, ω-3–enriched immunonutrition, and pre/pro/synbiotics—alone and in combination.
4. Intratumoral microbiota readouts: 16S/shotgun metagenomics with quantification of drug-modifying genes (e.g., cytidine deaminase) in resection tissue; exploratory circulating microbial DNA/metabolites as non-invasive biomarkers.
5. Methodologic rigor: Contamination control, pre-registered protocols, multiplicity-adjusted analyses.

By overlaying microbiome and metabolite features onto established inflammatory–nutritional indices (GNRI, PNI, CAR), the field can move toward a feasible, biomarker-guided model that personalizes both anticancer pharmacology and supportive nutrition to maximize adjuvant chemotherapy completion and clinical benefit in resectable PC.

## 5. Conclusions

This narrative review consolidates current evidence demonstrating that readily obtainable nutritional indices—including the GNRI, CAR, PNI, and sarcopenia—consistently predict completion of perioperative chemotherapy and long-term outcomes in resectable PC. These metrics, which integrate dimensions of nutritional reserve and systemic inflammation, capture key biological determinants of treatment tolerance and survival and are further modulated by gut microbiota–host interactions. The weight of both mechanistic and clinical data supports embedding early, standardized nutritional risk assessments into routine perioperative oncology workflows, followed by phase-specific, targeted interventions to preserve metabolic and functional capacity.

Despite robust retrospective evidence, prospective multicenter validation of these indices, ideally coupled with biomarker-guided stratification (e.g., inflammatory cytokine profiles and microbiota composition), is urgently needed. Such integration could transform nutritional management from a supportive adjunct to a proactive, central pillar of personalized oncologic care—enhancing chemotherapy adherence, reducing postoperative morbidity, and ultimately improving survival. By reframing perioperative nutrition as a modifiable, prognostically relevant domain, clinicians can leverage it as a therapeutic target rather than a secondary consideration.

## Figures and Tables

**Table 1:** Summary of nutritional assessment indices in pancreatic cancer care.

Summary of Nutritional Indices: Formulas and Clinical Use				
Index	Full name	Formula/Definition	Features	Clinical Significance
Sarcopenia		CT-based skeletal muscle mass at L3 (PMI, SMA)	Reflects muscle wasting and cachexia	Predicts AC intolerance and poor survival
GLIM	Global Leadership Initiative on Malnutrition	Phenotypic + Etiologic criteria (weight loss, BMI, muscle mass + intake/inflammation)	Global standardized malnutrition criteria	Linked to poorer survival and treatment adherence
BMI	Body Mass Index	Weight (kg)/Height (m)^2^	Simple indicator; does not reflect body composition	Limited use alone; may miss sarcopenic obesity
Albumin		Serum albumin level (g/dL)	Reflects nutrition and inflammation	Low levels predict poor prognosis and AC incompletion
PNI	Prognostic Nutritional Index	(10 × Albumin) + (0.005 × Lymphocyte count)	Integrates nutrition and immune status	Useful for predicting AC completion and prognosis
GNRI	Geriatric Nutritional Risk Index	(1.489 × Albumin) + (41.7 × BW/IBW)	Elderly specific nutritional risk index	Predicts AC adherence and survival outcomes
CAR	C-reactive Protein/Albumin Ratio	CRP/Albumin	Reflects inflammation and nutrition	Associated with AC failure and worse survival

AC: Adjuvant chemotherapy; Each index is defined by its key components, purpose, and clinical relevance. Parameters such as GNRI, PNI, CAR, and sarcopenia assessments play central roles in predicting chemotherapy tolerance and survival outcomes in resectable pancreatic cancer.

**Table 2:** Nutritional interventions during different treatment phases in pancreatic cancer and their intended clinical outcomes.

Nutritional Interventions and Therapeutic Impact							
Intervention	Target Phase	Purpose	Biomarkers /Triggers	Study Design/OCEBM	Endpoint (AC Completion)	Certainty (GRADE)	Representative Study
High-protein, high-calorie diet + ONS	Postoperative weeks 2–4; at AC start and during AC	Muscle maintenance, reduce toxicity	Low GNRI/PNI, low albumin, high CAR, low SMI	Prospective/retrospective pancreatic cancer cohorts; OCEBM 2–3	Improved completion (moderate)	Low	Gianotti et al. [32]
PERT	Early postoperative through entire AC course	Prevent malabsorption, weight loss	Clinical signs of PEI: weight loss, steatorrhea, malabsorption	Guideline-supported plus pancreatic cancer cohorts; OCEBM 2–3	Likely indirect improvement (strong)	Low	Bruno et al. [27]
Omega-3 fatty acids, immunonutrition	NAC through AC	Control inflammation; reduce toxicity	Elevated CRP/CAR; inflammatory phenotype	Mixed RCTs in GI cancers with pancreatic cancer subgroup analyses; OCEBM 2–3	Possible improvement	Very low–Low	Ueno et al. [59]
Resistance training/prehabilitation	NAC; postoperative weeks 2–4; during AC	Maintain or regain muscle and function	Low SMI; sarcopenia metrics	Small prospective/nonrandomized studies including pancreatic cancer; OCEBM 2–3	Signal of benefit (moderate)	Very low	Ngo-Huang et al. [63]
Probiotics/synbiotic	Postoperative; during AC	Improve gut milieu; lower infection/toxicity	Antibiotic exposure, diarrhea, dysbiosis	Postoperative RCTs exist, but data on AC completion are limited; OCEBM 2–3	Uncertain to possible	Very low	Nakaoka et al. [30]
Vitamin D analogs	NAC phase	Reduce fibrosis, enhance sensitivity	-	Preclinical/early-phase clinical; OCEBM 4–5	No direct evidence; effect	Very low	Sherman et al. [64]

## Data Availability

No new data were created or analyzed in this study. Data sharing does not apply to this article.

## Abbreviations

The following abbreviations are used in this manuscript:

AC	Adjuvant Chemotherapy
AE(s)	Adverse Event(s)
BMI	Body Mass Index
BTC	Biliary Tract Cancer
BW	Body Weight
CAR	C-reactive Protein-to-Albumin Ratio
ESPEN	European Society for Clinical Nutrition and Metabolism
GLIM	Global Leadership Initiative on Malnutrition
GNRI	Geriatric Nutritional Risk Index
GRADE	Grading of Recommendations, Assessment, Development and Evaluations
HDAC	Histone deacetylase
IL-6	interleukin-6
JAK	Janus kinase
NAC	Neoadjuvant Chemotherapy
NCCN	National Comprehensive Cancer Network
NF-κB	nuclear factor-κB
OCEBM	Oxford Centre for Evidence-Based Medicine
ONS(s)	Oral Nutritional Supplement(s)
OS	Overall Survival
PC	Pancreatic Cancer
PEI	Pancreatic Exocrine Insufficiency
PERT	Pancreatic Enzyme Replacement Therapy
PNI	Prognostic Nutritional Index
POC	Postoperative Complication
RDI	Relative Dose Intensity
SCFA	short-chain fatty acid
SMI	skeletal muscle index
STAT3	signal transducer and activator of transcription 3
TNF-α	tumor necrosis factor-α
